# Febrile Illness Associated with *Rickettsia conorii* Infection in Dogs from Sicily

**DOI:** 10.3201/eid1212.060326

**Published:** 2006-12

**Authors:** Laia Solano-Gallego, Linda Kidd, Michele Trotta, Marco Di Marco, Marco Caldin, Tommaso Furlanello, Edward Breitschwerdt

**Affiliations:** * Clinica e Laboratorio Veterinario Privato "San Marco," Padova, Italy;; † North Carolina State University, Raleigh, North Carolina, USA;; ‡ Clinica Veterinaria, Catania, Italy

**Keywords:** *Rickettsia conorii*, dog and illness, dispatach

## Abstract

We report serologic and molecular evidence of acute, febrile illness associated with *Rickettsia conorii* in 3 male Yorkshire terriers from Sicily (Italy).

Rickettsia conorii, transmitted by Ripicephalus sanguineus, causes Mediterranean spotted fever (MSF) in humans in Mediterranean countries, Sub Saharan Africa and Asia (*1*). Rickettsia spp. seroprevalence in dogs is high (26%–60%) in disease-endemic regions, and proximity to seroreactive dogs is a risk factor for MSF in humans (*2**,**3*). Recent studies reported the detection of Rickettsia DNA in the blood of European dogs (*4**,**5*). However, evidence that R. conorii infection causes illness in dogs is lacking (*2**,**3**,**6*). Illness has been associated with R. conorii natural infection in only 2 dogs since human MSF was described in 1932 (*6*). Moreover, the only clinical signs observed in experimentally infected dogs were pain, erythema, and edema at the injection site; and regional lymphadenopathy (*6*). We report infection with R. conorii ssp. conorii in 3 acutely ill, febrile Yorkshire terrier dogs, supported by PCR, DNA sequencing, and seroconversion.

## The Study

Between May and September 2005, three unrelated intact male Yorkshire terriers with a mean age of 4.3 years from Catania, Sicily, were brought to a local veterinarian; the dogs had the following histories: anorexia and lethargy of 2 days' duration (dog 1); anorexia, lethargy, and intermittent lameness of a few days' duration (dog 2); and intermittent vomiting, anorexia, and lethargy of a few days' duration (dog 3). Despite living mostly indoors, all 3 dogs had a recent history of tick exposure. All dogs had received current vaccination histories and had no history of serious illness. Results of the physical examination and hematologic, biochemical, and serum electrophoresis abnormalities at the time of onset of clinical signs and after 1 month (dogs 2 and 3) and 2 months (dog 1) of follow-up are provided in Table 1. Treatments instituted for all 3 dogs at onset of illness are described in Table 1.

**Table 1:** Clinical and laboratory data at the time of initial and follow-up examinations in 3 dogs with serologic and molecular evidence of natural *Rickettsia conorii* infection*

Date of evaluation, 2005	Physical examination abnormalities	Hematologic, biochemical, and serum electrophoresis abnormalities†	*Rickettsia* (16S and *ompA* gene) PCR
Dog 1			
May 31‡§	Fever (40.1°C), tachycardia, mildly enlarged right popliteal lymph node, blepharitis, hunched posture, stiff gait	Left shift neutrophilia (segmented 11,700; bands: 468) and thrombocytopenia (112). ALT (112), hypoproteinemia (5.0) and hypoalbuminemia (40.4). α_2_- (16.5) and β_1_-globulins (13.9)¶	Positive
Jun 8	No abnormalities	Mature neutrophilia (16,400); hyperglobulinemia (4.3); CRP (2.31); GGT (10.4); α_2_- (18.5), γ-globulins (23.3); hypoalbuminemia (40.9)	Negative
Aug 8	No abnormalities	No abnormalities	Negative
Dog 2			
Sep 19‡§	Fever (41°C), ptyalism, joint pain, lameness in right rear limb	Microcytic-hyperchromic anemia (MCHC 63.7; MCV 56; Hct 25.3); mature neutrophilia (11,680)¶	Positive
Sep 28	No abnormalities	Hyperglobulinemia (4.3);­ CRP (0.64); α_2_- (17.5), and γ-globulin (24.6); hypoalbuminemia (41.1)	Negative
Oct 25	No abnormalities	No abnormalities	Negative
Dog 3			
Sep 17§#	Fever (41°C), abdominal pain, dehydration, peripheral lymphadenomegaly (popliteal and prescapular lymph nodes), conjunctivitis	Thrombocytopenia (69); hypoproteinemia (5.3); hypoalbuminemia (28.6); α_2_- (14.8), β_1_- (13.0), β_2_- (15.6), γ-globulins (23.0)¶	Positive
Sep 23‡	No abnormalities	Lymphocytosis (5,594); hyperglobulinemia (4.1); CRP (2.7); BUN (75); α_2_-globulin (19.3); hypoalbuminemia (44.9)	Negative
Oct 25	No abnormalities	↑BUN (54)	Negative

*ALT, alanine aminotransferase; CRP, C-reactive protein; GGT, γ-glutamyl transferase; MCHC, mean cell hemoglobin concentration; MCV, mean cell volume; Hct, hematocrit; BUN, blood urea nitrogen; ↑­, increase. †Reference interval: Hct 38.6%–54.5%; MCV 61–72 fL; MCHC 34–38 g/dL; segmented neutrophils 3,800–8,800/μL; bands neutrophils 0–300/μL; lymphocytes 1,300–4,100/μL; platelets 160–440 × 10^3^/μL); total protein 5.5–7.5 g/dL; globulins 2.6–4.0 g/dL; albumin 53%–65%; α_2_-globulins 8.0%–14.0%; β_1_-globulins 2.0%–5.0%; β_2_-globulins 3.0%–9.0 %; γ-globulins 6.0%–15.0%); BUN 18–43 mg/dL; CRP 0.0–0.15 mg/dL; ALT 15–65 IU/L; GGT 2.0–8.0 IU/L. ‡Treatment with doxycycline (10 mg/kg/once a day by mouth/1 month) was started. §Intravenous fluids were administered. ¶CRP was not measured. #Treatment with ceftriaxone (30 mg/kg/twice a day intravenously/for 5 days) was started.

EDTA-blood and serum samples were obtained by the attending veterinarian at the time of clinical assessment (before treatment), then 1 week later and 1 month (dogs 2 and 3) or 2 months later (dog 1). DNA extraction was performed from whole blood samples (*5**,**7*). A quantitative PCR (qPCR) for detection of Rickettsia spp., Anaplasma phagocytophilum, Ehrlichia canis, and Leishmania infantum in DNA samples was performed by using a Light Cycler (Roche, Mannheim, Germany). PCR amplification was carried out with Rickettsia (Rr-prim3 5´-GAAACCGAAAGAGAATCTTCCGAT-3´ and Rr-prim4 5´-TCCTAGTGTAGAGGTGAAATTCTTA-3´ [8]), E. canis, A. phagocytophilum (fragment of 16S rRNA gene), and L. infantum LCSet primers and probes following manufacturer's instructions (TIB Molbiol, Centro Biotecnologie Avanzate, Genova, Italy) (*5**,**7*). Conventional Babesia genus PCR was performed (*9*). Borrelia burgdorferi sensu lato qPCR was performed by a commercial laboratory (http://www.scanelis.com). PCR results for all infectious agents listed above, with the exception of Rickettsia, were negative in all dogs.

PCRs for Rickettsia that use the outer membrane protein A (ompA) gene to amplify 632 bp (*10*) and 212 bp (107F 5´-GCTTTATTCACCACCTCAAC-3´ and 299R 5´-TRATCACCACCGTAAGTAAAT-3´) (*7*) amplicons were performed. For dog 1, a 632-bp amplicon was cloned by using the TOPO TA Cloning (Invitrogen, Carlsbad, CA, USA) and sequenced (GenBank accession no. DQ518245) (*7*). For dogs 2 and 3, a 212-bp amplicon was subjected to direct sequencing (accession no. DQ518246, DQ518247) (*7*). PCR results are summarized in Table 1.

Consensus sequences were aligned [(BIOEDIT version 7.0 (ClustalW)] with known sequences in GenBank using the basic local alignment search tool (BLAST; available from http://www.ncbi.nlm.nih.gov/BLAST/). The sequence obtained from all 3 dogs was 100% homologous to a portion of the complete genome sequence corresponding to the ompA gene from R. conorii (Malish 7, accession no. AE008674).

Immunofluorescent assays to detect antibodies to R. rickettsii, R. conorii, B. burgdorferi sensu stricto, E. canis, Babesia canis, A. phagocytophilum, L. infantum, Bartonella henselae, and B. vinsonii ssp. berkhoffi antigens were performed (*3**,**7*). Results are presented in Table 2.

**Table 2:** Reciprocal IFA titers for the 3 dogs with clinical and molecular evidence of natural *Rickettsia conorii* infection*

Dog no.	Date, 2005 (days after clinical signs)	*R. conorii*		*R. rickettsii*		*Anaplasma phagocytophilum*	*Borrelia burgdorferi*
		IgM	IgG	IgM	IgG	IgG	IgG
1	Jun 8 (7)	1:1,280	1:20,480	1:1,280	1:20,480	1:160	Neg
1	Aug 3 (65)	1:80	1:320	1:80	1:320	Neg	1:160
2	Sep 19 (0)	1:640	1:80	1:640	1:80	Neg	Neg
2	Sep 28 (9)	1:1,280	1:40,960	1:640	1:20,480	Neg	Neg
2	Oct 25 (37)	1:640	1:10,240	1:640	1:5,120	Neg	Neg
3	Sep 17 (0)	1:1,280	1:80	1:2,560	Neg	Neg	Neg
3	Sep 23 (6)	1:10,240	1:10,240	1:10,240	1:5,120	Neg	Neg
3	Oct 25 (39)	1:2,560	1:5,120	1:5,120	1:5,120	Neg	Neg

*IFA, immunofluorescence assay; Ig, immunoglobulin; Neg, negative. †All dogs were seronegative for *L. infantum*, *E. canis*, *B. canis*, *B. henselae*, and *B. vinsonii* ssp. berkhoffi at all time points.

## Conclusions

Clinicopathologic abnormalities detected in these dogs at initial examination, including acute onset of fever, lethargy, thrombocytopenia, anemia, mildly increased liver enzyme activities and hypoalbuminemia, were very similar to abnormalities associated with spotted fever group (SFG) rickettsioses in dogs and humans (*1*). In addition, R. conorii DNA was amplified in all dogs during the acute illness. Further evidence for R. conorii infection as a cause of the associated clinical signs was provided by the subsequent failure to detect DNA in dogs 1 and 2, 1 week after treatment with doxycycline and the rapid resolution of clinical signs 2 days after initiating doxycycline therapy. Clinical signs in dog 3 resolved in 4 days, while the dog was receiving ceftriaxone, which has no known anti-rickettsial efficacy (*1*). Spontaneous immune clearance of R. conorii likely accounted for the resolution of clinical signs in dog 3 (*6*).

The 4-fold increase in IgG antibody titers in dogs 2 and 3 supports seroconversion, which is consistent with an acute R. conorii infection (*11*). Additionally, the initially high IgM titer in dog 1 after the onset of illness compared with a much lower IgM titer after 65 days is also supportive of an acute infection and is consistent with observations of human serologic test results (*1*). IgM titers rise rapidly and then disappear by day 35 and 80 in dogs experimentally infected with R. conorii and R. rickettsii, respectively (*6**,**11*). However, high R. rickettsii IgM titers are detected in dogs that do not seroconvert, based upon IgG antibodies (*11*). Thus, the presence of IgM supports but does not prove acute SFG infection in dogs.

Coinfection with A. phagocytophilum or B. burgdorferi could have contributed to clinical signs observed in dog 1. This dog had a low serum A. phagocytophilum titer 7 days after initial examination and also seroconverted to B. burgdorferi. A. phagocytophilum causes an acute febrile illness in dogs and humans, similar to the findings described here (*12*). B. burgdorferi does not cause clinical signs in dogs until 60–150 days after experimental infection (*13*); therefore, despite seroconversion, the acute clinical signs in dog 1 were not likely to have been related to B. burgdorferi infection. Moreover, PCR amplification of DNA from organisms other than R. conorii was not found in any dog.

All dogs were intact, male, genetically unrelated Yorkshire terriers. Although an increased risk for Rocky Mountain spotted fever has not been reported in Yorkshire terriers, purebred dogs infected with R. rickettsii appear to be more prone to clinical illness (*14*). Notably, this breed seems to be at increased risk for Babesia canis infection (*15*). Male dogs and men may be at increased risk for infection and may develop more severe illness with R. rickettsii and R. conorii (*1**,**14*), and male dogs are more likely to be R. conorii seroreactive (*3*). It has been suggested that more severe illness may develop in English springer spaniels with suspected phosphofructokinase deficiency and persons with glucose 6-phosphate dehydrogenase deficiency when infected with R. rickettsii and R. conorii (*1**,**14*). Although inherited immunodeficiencies have not been reported in Yorkshire terriers, and all dogs were previously healthy, an inherited metabolic or immunologic defect cannot be ruled out because specific testing was not performed.

Although a metabolic or immunologic defect may be necessary for illness to develop in dogs of various breeds after R. conorii infection, other potential explanations can be made for the discrepancy between high R. conorii seroprevalence rates among healthy dogs and lack of reports of clinical illness. The high R. conorii seroprevalence in healthy dog populations suggests that exposure to SFG rickettsiae is common. However, the acute, nonspecific, and potentially self-limiting nature of R. conorii infection, combined with a low index of suspicion by regional veterinarians and a historical lack of specific diagnostic techniques, may have precluded the prior association of clinical signs with R. conorii infection in dogs. Further evidence should be gathered regarding the extent to which R. conorii causes clinical disease in dogs.
